# Pressure Signal Enhancement of Slowly Increasing Leaks Using Digital Compensator Based on Acoustic Sensor

**DOI:** 10.3390/s19194317

**Published:** 2019-10-05

**Authors:** Fang Wang, Weiguo Lin, Zheng Liu, Xianbo Qiu

**Affiliations:** 1 College of Information Science and Technology, Beijing University of Chemical Technology, Beijing 100029, China; 2016400136@mail.buct.edu.cn (F.W.); xbqiu@mail.buct.edu.cn (X.Q.); 2 Faculty of Applied Science, University of British Columbia Okanagan, Kelowna, BC V1V 1V7, Canada; zheng.liu@ubc.ca

**Keywords:** leak detection, pressure signal, digital compensator, slowly increasing leak

## Abstract

Pipeline leak detection technologies are critical for the safety protection of pipeline transportation. However, they are insensitive to slowly increasing leaks. Therefore, this study proposes an enhancement method for slowly increasing leak signals. By analyzing the characteristics of pressure signals of slowly increasing leaks, a digital compensator is developed to overcome the disadvantages of pressure signals and enhance the pressure signals. According to the frequency response analysis of the digital compensator, the enhancement principle is the parameter adjustment of the digital compensator. Therefore, this paper further proposes an adaptive adjustment method of the parameter to enhance different degrees of leak signals online in real-time, and the proposed method is evaluated using two field pipelines. The experimental results demonstrate that this method is suitable not only for enhancing slowly increasing leaks but also for enhancing abrupt leaks.

## 1. Introduction

Many pipelines, such as chemical feedstock transportation pipelines, urban gas pipelines, and water pipelines, suffer from leaks which may occur because of corrosion, aging pipelines, or third party damages [1,2,3,4,5]. Nowadays, researchers have developed many leak monitoring systems which mainly have three steps: (1) sensing signals through sensors, (2) leak diagnosis, and (3) leak location. Therefore, the effectiveness of leak signals is crucial for pipeline leak detection.

When a pipeline leak occurs, the leak of the medium will cause many physical changes, such as the pressure, strain, acoustic wave. For the heated medium, the temperature would also vary. These physical variables can be measured by the sensors installed on the pipeline. Moreover, the most widely used sensors are the optical fiber sensors [6,7], acoustic transducers [8,9,10,11] and pressure transducers [12,13,14,15]. The fiber optical sensors can sense leak signals by monitoring temperature, strain, or acoustic signals based on laying a long sensing cable along the outside of a pipeline [7,16,17]. This method has been widely applied in pipeline leak detection thanks to its high sensitivity and reliability [16,18]. However, the laying and maintenance of fiber optical sensors are often costly and difficult, especially for underground pipelines.

The acoustic transducers are installed on both ends of the pipeline and can monitor a pipeline leak by measuring relative pressure [19]. As presented in [20,21], the acoustic transducer has a wide frequency response and high sensitivity. Moreover, the waveform of the acoustic signal is sharp, which helps the accurate location of a leak. Therefore, the acoustic transducer is widely employed in pipeline leak detection [22,23,24,25]. However, the acoustic transducers cannot sense slowly increasing leaks. In this paper, pipeline leaks are divided into abrupt and slowly increasing leaks according to whether the leak aperture changes slowly. It is important to note that the slowly increasing leak is not the same as the small leak.

Compared with acoustic transducers, pressure transducers are used to measure absolute pressure. Furthermore, almost all pipelines have been equipped with pressure transducers due to their stability and low cost. However, the pressure changes caused by the leak are too small in contrast to the pressure fluctuation and whole pressure transducer range [12,19,26], particularly for slowly increasing leaks. Additionally, the infection point of a slowly increasing leak signal is difficult to obtain accurately, which will influence the precision of the leak location. Nevertheless, the pressure signal can still record the pressure drop process caused by a slowly increasing leak, which provides the possibility of detecting the slowly increasing leak.

Therefore, after taking into account the cost and ability to measure slowly increasing leaks, this paper designs a digital compensator based on the advantages of the acoustic transducer to enhance pressure signals of slowly increasing leaks. Moreover, the input and output of the digital compensator are the pressure signals and the compensated signals, respectively. According to the frequency response of the digital compensator, the pressure signals of slowly increasing leaks can be enhanced by adjusting the parameter (the discretization frequency $f_{s}$) of the digital compensator. Hence, a parameter adaptive adjustment method is proposed to real-time online enhance pressure signals of with different degrees of leaks.

The rest of this paper is organized as follows. Section 2 describes the methodology including the development and the stability analysis of the digital compensator, the signal enhancement principle of slowly increasing leaks, the adaptive adjustment of parameters, and the leak detection and location. Section 3 describes the field experiments. The conclusions of this study are summarized in Section 4.

## 2. Methodology

**Figure 1:**
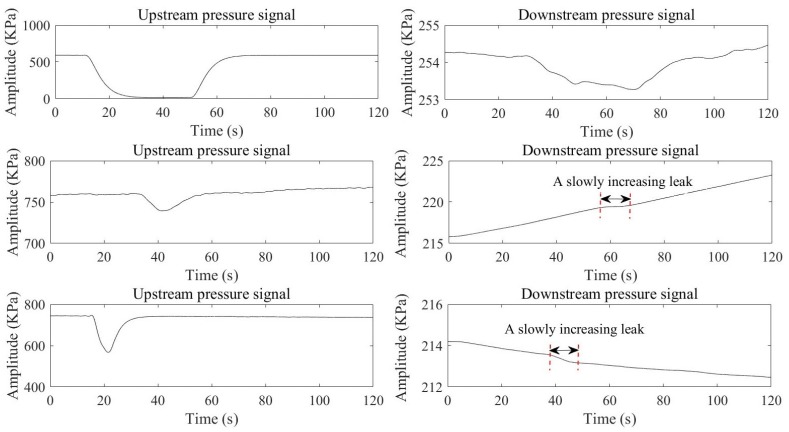
Typical slowly increasing leak signals.

In practice, slowly increasing leaks may occur during the pipeline pressure regulation process (increase, decrease, or fluctuation). Figure 1 shows some instances of slowly increasing leaks coming from the pressure transducers installed on the crude oil pipeline. Moreover, the specific parameters of the field pipeline are given in Section 3. As can be seen, leak pressures are highly susceptible to the static pressure of the pipeline, and their waveforms are smoother and milder. Moreover, these characteristics make slowly increasing leaks hard to identify. Consequently, the purpose of the digital compensator is to compensate for these characteristics and enhance pressure signals.

**Figure 2:**
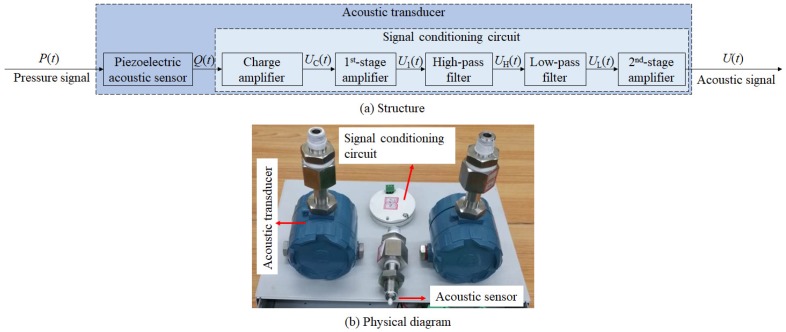
Acoustic transducer: (**a**) structure, (**b**) physical diagram.

As the analysis in Section 1, the acoustic and pressure transducers have a certain complementarity. Therefore, the digital compensator is developed based on the acoustic transducer. Figure 2 shows the structure and physical diagram of the acoustic transducer that is composed of a piezoelectric acoustic sensor and signal conditioning circuit.

### 2.1. Development of the Digital Compensator

**Figure 3:**
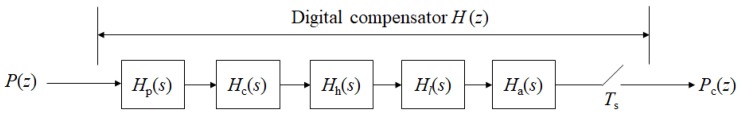
Structure of the digital compensator.

**Figure 4:**
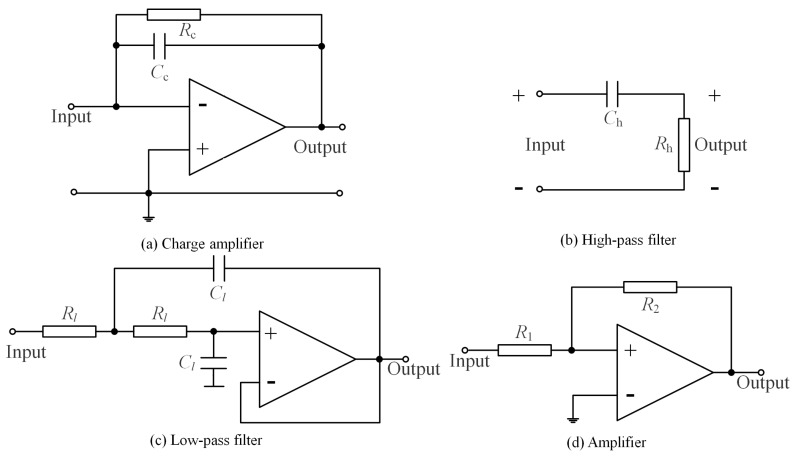
Analog circuit diagram: (**a**) charger amplifier, (**b**) high-pass filter, (**c**) low-pass filter, (**d**) amplifier.

Based on the acoustic transducer (Figure 2), the structure of the digital compensator is shown in Figure 3, where the input P(z) is the original pressure signal from the pressure transducer and the output $P_{c}\left(z\right)$ is the compensated signal. Moreover, the $H_{p}\left(s\right)$, $H_{c}\left(s\right)$, $H_{h}\left(s\right)$, $H_{l}\left(s\right)$, and $H_{a}\left(s\right)$ are the transfer functions of the piezoelectric acoustic sensor, charge amplifier (Figure 4a), first-order high-pass filter (Figure 4b), second-order low-pass filter (Figure 4c) and amplifier (Figure 4d), respectively.

Since the internal parameters and structure of the piezoelectric acoustic sensors produced by different manufacturers vary widely, and these specific data are confidential to the customer, it is impossible to establish a corresponding mechanism model based on the structure of the sensor. The ideal operating state of a senor is the linear relationship between input and output. Moreover, for the customer of the sensor, the known parameters are its sensitivity and range. Based on this, the $H_{p}\left(s\right)$ is expressed as:

(1) $$H_{p}\left(s\right)=A_{p}$$

where $A_{p}$ is the sensitivity coefficient of the piezoelectric acoustic sensor. Moreover, according to the principle of analog circuit shown in Figure 4, the $H_{c}\left(s\right)$, $H_{h}\left(s\right)$, $H_{l}\left(s\right)$, and $H_{a}\left(s\right)$ are:

(2) $$H_{c}\left(s\right)=\frac{-R_{c}s}{\tau_{c}s+1}$$

(3) $$H_{h}\left(s\right)=\frac{{\left(\tau_{h}s\right)}^{2}}{{\left(\tau_{h}s\right)}^{2}+2\tau_{h}s+1}$$

(4) $$H_{l}\left(s\right)=\frac{1}{{\left(\tau_{l}s\right)}^{2}+2\tau_{l}s+1}$$

(5) $$H_{a}\left(s\right)=A_{a}$$

where $R_{c}$ is the feedback resistance of the charge amplifier; $\tau_{c}$, $\tau_{h}$, and $\tau_{l}$ are the time constant of the charge amplifier, high- and low-pass filters; $A_{a}$ is the gain of the amplifier. Hence, the continuous model H(s) is:

(6) $$H\left(s\right)=H_{p}\left(s\right)H_{c}\left(s\right)H_{h}\left(s\right)H_{l}\left(s\right)H_{a}\left(s\right)=\frac{-A_{p}A_{a}R_{c}}{\tau_{c}\tau_{l}^{2}}\frac{s^{2}}{\prod_{m=1}^{4}\left(s+1/\tau_{m}\right)}$$

where $\tau_{1}=\tau_{c},\tau_{2}=\tau_{h}$, and $\tau_{3}=\tau_{4}=\tau_{l}$. Then, a bilinear transformation method is used to discretize the continuous model H(s). Moreover, the discrete model H(z) can be expressed as:

(7) $$H\left(z\right)=-4A\tau_{h}f_{s}^{2}\frac{{\left(z-1\right)}^{2}{\left(z+1\right)}^{2}}{\prod_{m=1}^{4}\left(2f_{s}\tau_{m}+1\right)\left(z-\frac{2f_{s}\tau_{m}-1}{2f_{s}\tau_{m}+1}\right)}$$

where $A=A_{p}A_{a}R_{c}$; $f_{s}$ is the discretization frequency, and it has the same definition with the sampling frequency. According to the *Z* inverse transformation of Equation (7), the backward difference equation is:

(8) $$\sum_{m=0}^{4}a_{m}P_{c}\left(k-m\right)=\sum_{n=0}^{4}b_{n}P\left(k-n\right)$$

where *k* represents the sampling time, and the values of the coefficients $a_{m}$ and $b_{n}$ can be calculated with Equation (9).

(9) $$\left[\begin{array}{cc} a_{0} & b_{0}\\ a_{1} & b_{1}\\ a_{2} & b_{2}\\ a_{3} & b_{3}\\ a_{4} & b_{4} \end{array}\right]=\left[\begin{array}{cc} 1 & -4A\tau_{h}f_{s}^{2}\\ -(q_{c}+q_{h}+2q_{l}) & 0\\ q_{l}^{2}+2q_{l}\left(q_{c}+q_{h}\right)+q_{c}q_{h} & 8A\tau_{h}f_{s}^{2}\\ -q_{l}\left(q_{c}q_{l}+q_{l}q_{h}+2q_{c}q_{h}\right) & 0\\ q_{c}q_{h}q_{l}^{2} & -4A\tau_{h}f_{s}^{2} \end{array}\right],\left\{\begin{array}{c} q_{c}=\frac{2f_{s}\tau_{c}-1}{2f_{s}\tau_{c}+1}\\ q_{h}=\frac{2f_{s}\tau_{h}-1}{2f_{s}\tau_{h}+1}\\ q_{l}=\frac{2f_{s}\tau_{l}-1}{2f_{s}\tau_{l}+1} \end{array}\right.$$

So far, the digital compensator is established. Moreover, its input and output are the original pressure signal P(k) and the compensated signal $P_{c}\left(k\right)$, respectively. Equations (6)–(8) are its continuous, discrete, and backward difference forms.

### 2.2. Stability Analysis of the Digital Compensator

According to Equation (7), the poles of the digital compensator H(z) are:

(10) $$p_{i}=\frac{2f_{s}\tau_{i}-1}{2f_{s}\tau_{i}+1}$$

where i=1,2,3,4; $\tau_{1}=\tau_{c},\tau_{2}=\tau_{h}$, and $\tau_{3}=\tau_{4}=\tau_{l}$. As the discretization frequency $f_{s}$ and the time constant $\tau_{i}$ are both positive, then:

(11) $$\lim_{f_{s}\to 0}\left(2f_{s}\tau_{i}\right)=0\;\mathrm{and}\;2f_{s}\tau_{i}-1<2f_{s}\tau_{i}+1$$

Hence, the pole $p_{i}$ satisfies:

(12) $$\left|p_{i}\right|=\left|\frac{2f_{s}\tau_{i}-1}{2f_{s}\tau_{i}+1}\right|<1$$

According to Equation (12), the digital compensator is stable with $f_{s}>0$ and $\tau_{i}>0$.

### 2.3. Pressure Signal Enhancement Principle of Slowly Increasing Leaks

The frequency response $H(e^{j\omega })$ of the digital compensator H(z) (Equation (7)) is:

(13) $$H\left(e^{j\omega }\right)=H\left(z\right){|}_{z=e^{j\omega }}=-4A\tau_{h}f_{s}^{2}\frac{{\left(e^{j\omega }-1\right)}^{2}{\left(e^{j\omega }+1\right)}^{2}}{\prod_{m=1}^{4}\left(2f_{s}\tau_{m}+1\right)\left(e^{j\omega }-\frac{2f_{s}\tau_{m}-1}{2f_{s}\tau_{m}+1}\right)}$$

According to Euler’s formula ($e^{j\omega }=\cos\omega +j\sin\omega$), Equation (13) can be rewritten as:

(14) $$\begin{array}{ll} H(e^{j\omega }) & =-4A\tau_{h}f_{s}^{2}\frac{{\left(\cos\omega -1+j\sin\omega \right)}^{2}{\left(\cos\omega +1+j\sin\omega \right)}^{2}}{\prod_{m=1}^{4}\left(2f_{s}\tau_{m}+1\right)\left(\cos\omega -\frac{2f_{s}\tau_{m}-1}{2f_{s}\tau_{m}+1}+j\sin\omega \right)}\\ & =-16A\tau_{h}f_{s}^{2}\sin^{2}\omega \frac{{\left(-\sin\omega +j\cos\omega \right)}^{2}}{\prod_{m=1}^{4}\left(2f_{s}\tau_{m}+1\right)\left(\cos\omega -\frac{2f_{s}\tau_{m}-1}{2f_{s}\tau_{m}+1}+j\sin\omega \right)} \end{array}$$

Hence, the amplitude response $\left|H(e^{j\omega })\right|$ (the specific derivation is given in Appendix A) is:

(15) $$\begin{array}{ll} \left|H(e^{j\omega })\right| & =20\mathrm{lg}\left(A\tau_{c}\tau_{h}^{2}\right)+10\mathrm{lg}\frac{2f_{s}\tau_{c}\tau_{h}\tau_{l}\tan\frac{\omega }{2}}{\tau_{h}^{2}\tau_{l}^{2}+{\left(2f_{s}\tau_{c}\tau_{h}\tau_{l}\tan\frac{\omega }{2}\right)}^{2}}\\ & +10\mathrm{lg}\frac{2f_{s}\tau_{c}\tau_{h}\tau_{l}\tan\frac{\omega }{2}}{\tau_{c}^{2}\tau_{l}^{2}+{\left(2f_{s}\tau_{c}\tau_{h}\tau_{l}\tan\frac{\omega }{2}\right)}^{2}}+20\mathrm{lg}\frac{2f_{s}\tau_{c}\tau_{h}\tau_{l}\tan\frac{\omega }{2}}{\tau_{c}^{2}\tau_{h}^{2}+{\left(2f_{s}\tau_{c}\tau_{h}\tau_{l}\tan\frac{\omega }{2}\right)}^{2}} \end{array}$$

According to Equation (15), it can be concluded that: (1) the $f_{s}$ is the scale factor, (2) *A* is the translation step, and (3) $\tau_{c}$, $\tau_{h}$, and $\tau_{l}$ have both scale and translational effects.

**Figure 5:**
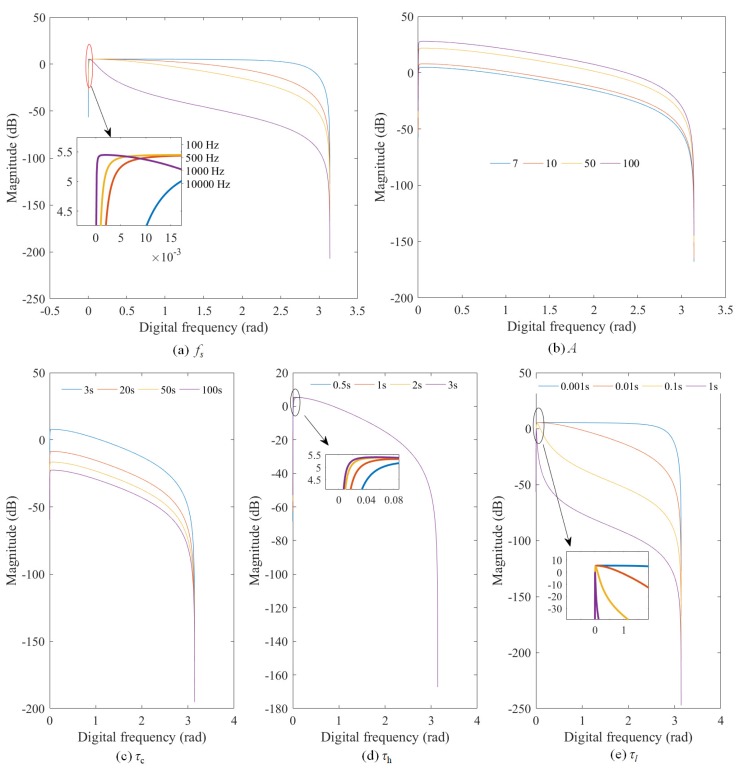
Waveforms of $\left|H(e^{j\omega })\right|$ with different parameters: (**a**) $f_{s}$, (**b**) *A*, (**c**) $\tau_{c}$, (**d**) $\tau_{h}$, (**e**) $\tau_{l}$.

Figure 5 shows the amplitude response $\left|H(e^{j\omega })\right|$ varying with the parameters ($f_{s}$, *A*, $\tau_{c}$, $\tau_{h}$, and $\tau_{l}$) and the corresponding cutoff frequency is given in Table 1. It is important to note that: (1) when a parameter is a variable, other parameters are set to fixed values ($f_{s}$ = 100 Hz, $\tau_{c}$ = 4 s, $\tau_{h}$ = 2 s, $\tau_{l}$ = 0.01 s and $A=A_{p}A_{a}R_{c}$ = 5.348 × 10${}^{-11}$ C/Pa × 3 × 2 × 10${}^{10}$ Ω = 3.21 Ω×C/Pa); and (2) according to the structure of the digital compensator, the cutoff frequency of the charge amplifier, high-pass filter and low-pass filter should be $f_{c}<f_{h}<f_{l}$, since $f={\left(2\pi \tau \right)}^{-1}$, the change of $\tau_{c}$, $\tau_{h}$ and $\tau_{l}$ needs to satisfy $\tau_{c}>\tau_{h}>\tau_{l}$. It can be seen from Figure 5 and Table 1 that the parameters $f_{s}$, $\tau_{c}$, $\tau_{h}$, and $\tau_{l}$ play a telescopic effect on the amplitude response $\left|H(e^{j\omega })\right|$, in which the effect of $f_{s}$ and $\tau_{l}$ is obvious.

Based on the above analysis, the amplitude response $\left|H(e^{j\omega })\right|$ changes with the frequency, that is, the digital compensator |H(z)| can selectively pass and amplify certain frequencies of the pressure signal. Therefore, the digital compensator has a frequency selective characteristic, and these passing frequencies are determined by the parameters $f_{s}$, $\tau_{c}$, $\tau_{h}$, and $\tau_{l}$.

**Figure 6:**
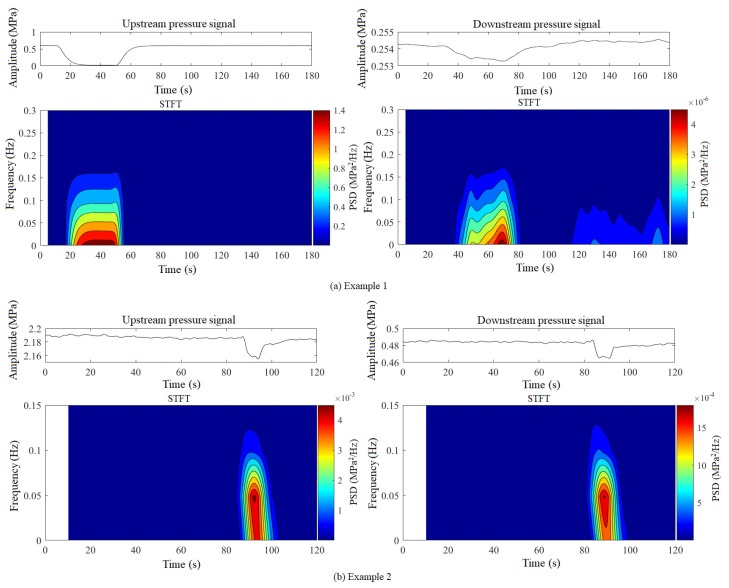
Frequency distributions of leak pressure signals: (**a**) example 1, (**b**) example 2.

Different leak pressure signals have different frequency distributions, as illustrated in Figure 6. Therefore, in order to enhance pressure signals, the pass-band of the digital compensator should be matched or overlapped with the frequency band of the leak pressure signals, which can be achieved by adaptively adjusting the parameters $f_{s}$, $\tau_{c}$, $\tau_{h}$, and $\tau_{l}$ of the digital compensator. Since the adjustment of the $\tau_{c}$, $\tau_{h}$, and $\tau_{l}$ needs to satisfy $\tau_{c}>\tau_{h}>\tau_{l}$, this study adjusts only the discretization frequency $f_{s}$.

To sum up, the key to the enhancement of slowly increasing leaks is that the digital compensator has suitable parameters.

### 2.4. Adaptive Adjustment of the Discretization Frequency $f_{s}$

The discretization frequency $f_{s}$ should satisfy Nyquist sampling theorem:

(16) $$f_{s}\geq 2f_{\max}$$

where $f_{\max}$ is the maximum frequency of the signal. Since the sampling frequency *f* of pressure signals have satisfied this theorem, the $f_{s}$ can be selected from the values greater than the *f* ($f_{s}\geq f$).

**Figure 7:**
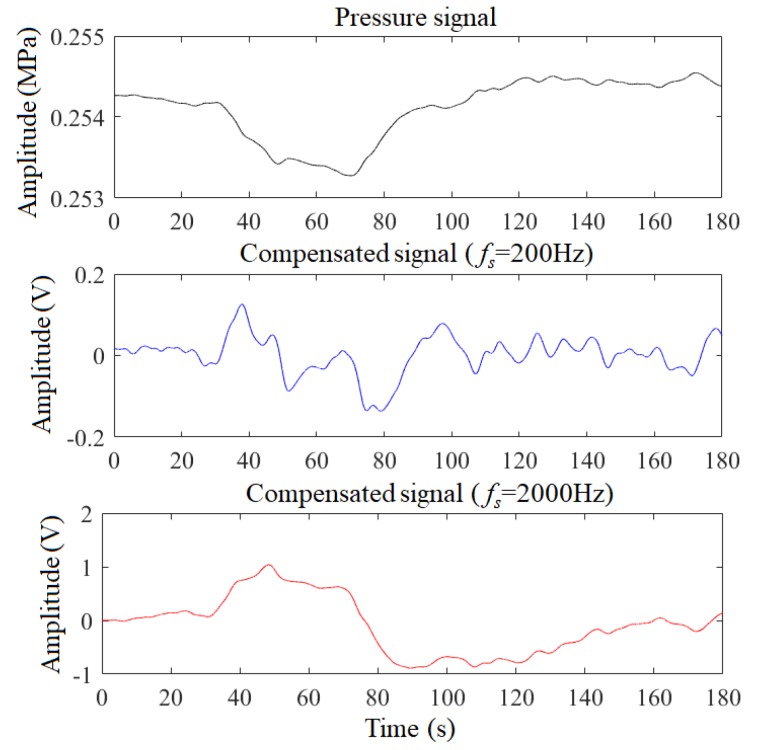
Compensated signals with different discretization frequency $f_{s}$.

Figure 7 shows the time-domain waveforms of compensated signals with different discretization frequency $f_{s}$. When $f_{s}$ = 2000 Hz, the waveform of the compensated signal almost reproduces the pressure signal except for the amplitude and polarity. Therefore, when $f_{s}$ increases to a certain extent, the digital compensator has almost no selectivity for the pressure signal, that is the corresponding compensated signal is not enhanced. At this time, the width of the leak waveform in the compensated signal is approximate to the width in the pressure signal. And the better performing parameters should make the former smaller than the latter, as the $f_{s}$ = 200 Hz shown in Figure 7.

**Figure 8:**
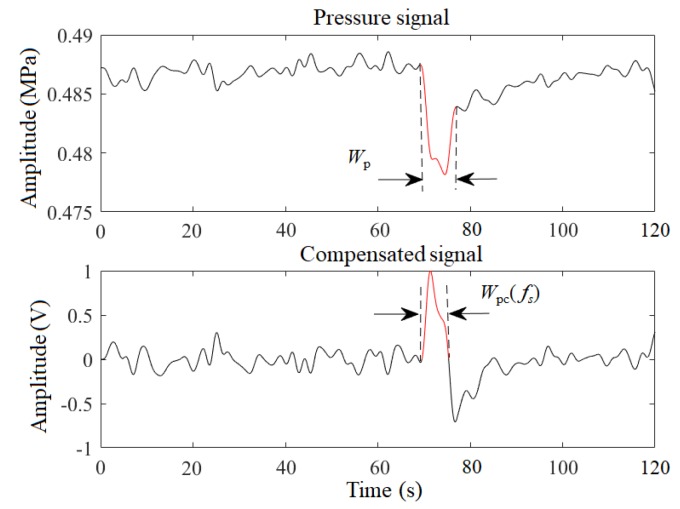
Width characteristic.

According to the above analysis, the width characteristic of the signal can further constrain the range of the $f_{s}$. Based on the signal decomposition method [27], a compensated signal is decomposed into sub-signals, and the sub-signal width of the maximum peak is taken as its width characteristic which is denoted as $W_{P_{c}}\left(f_{s}\right)$, as shown in Figure 8. Similarly, the pressure signal is divided into intervals based on extreme points. The width between the largest drop interval and the adjacent ascending interval in the pressure signal is taken as the width characteristic of the pressure signal and is denoted as $W_{P}$, as shown in Figure 8. Then, the $f_{s}$ should make the $W_{P_{c}}\left(f_{s}\right)$ smaller than the $W_{P}$. Therefore, the constraints of parameter $f_{s}$ are

(17) $$s.t.\;f_{s}\geq f\;\mathrm{and}\;W_{P_{c}}\left(f_{s}\right)>W_{P}$$

The signal-to-noise ratio (SNR) of the compensated signal is an indicator that can evaluate whether the pressure signal is enhanced. However, the SNR calculation depends on the signal abnormality, which is unknown before leak detection [27]. Therefore, it is not feasible to directly use SNR as an indicator to select an appropriate $f_{s}$. Moreover, the better performing compensated signal has two characteristics: (1) its leak amplitude is significantly larger than the normal, and (2) it is evenly distributed around zero. The former can be described by the standard deviation, and the latter can be represented by the number of intervals in which the signal can be decomposed by its zero crossings. Hence, this paper proposes the optimization goal shown below:

(18) maxJ=cσ+(1−c)M

where σ is the standard deviation of the normalized signal; *M* is the number of intervals [27]; *c* is the penalty factor and is determined by:

(19) $$c=\frac{1}{N}\left[n_{e}\left(i\right)-n_{s}{\left(i\right)|}_{\max\left\{P\left[n_{e}\left(i\right)\right]-P\left[n_{s}\left(i\right)\right]\right\}}\right]$$

where *N* is the data length of the signal; $n_{e}\left(i\right)$ is the end position of the *i*th drop interval; and $n_{s}\left(i\right)$ is the start position of the *i*th drop interval.

**Figure 9:**
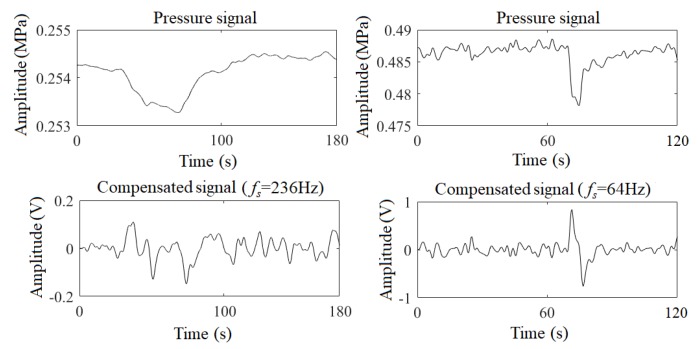
Pressure signals and compensated signals with the optimized $f_{s}$.

Therefore, under the constraint shown in Equation (17), the optimized $f_{s}$ should make the optimization goal maximize. Figure 9 shows the original pressure signal and the compensated signals with the optimized $f_{s}$, where *c* is 0.221 and 0.044, respectively. As can be seen, the waveforms of the compensated signals are sharp, and the infection points are obvious. Therefore, the pressure signals are enhanced by the digital compensator with suitable parameters.

### 2.5. Leak Detection and Location

After the signal enhancement, these signals should be judged whether they are leak signals, which can be achieved by signal conditioning or machine learning. This paper uses the leak detection method based on model-free abnormal acoustic signal isolation [27], and the equation of leak location is [27]

(20) $$x_{L}=\frac{1}{2}\left(L+a\Delta t\right)$$

where $x_{L}$ is the distance from the leak point to the upstream sensor; *a* is the velocity of signals propagating inside the pipeline; *L* is the length between the upstream and downstream sensors; and Δt is the time difference of the upstream and downstream leak signals, which can be calculated by Equations (21)–(23).

(21) $$R_{xy}\left(\Delta n\right)=\lim_{N\to +\infty }\frac{1}{N}\sum_{i=1}^{N}x\left(i\right)y\left(i+\Delta n\right)$$

(22) $$R_{xy}\left(\Delta n_{0}\right)=\max\;R_{xy}\left(\Delta n\right)$$

(23) $$\Delta t=T\times \Delta n_{0}$$

where $R_{xy}$ is the cross-correlation coefficient; Δn is the delay points, $\Delta n_{0}$ is the delay points corresponding to the maximum of the cross-correlation coefficient $R_{xy}$; *N* is the data length of a signal; x(i) is the upstream signal; and y(i) is the downstream signal.

## 3. Field Experiments

**Figure 10:**
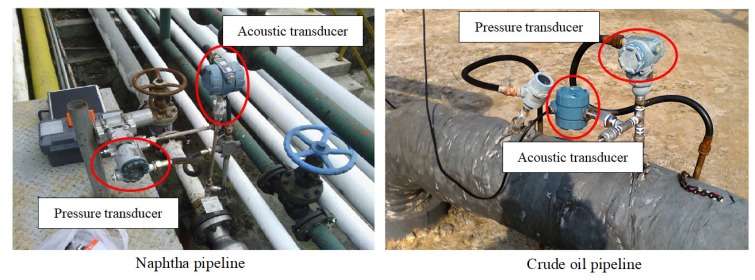
The installation of transducers on pipelines.

In order to verify the effectiveness of the method based on the digital compensator, two field pipelines (a naphtha pipeline and a crude oil pipeline) are used. Figure 10 shows the installation diagram of the transducers on field pipelines. Their parameters are presented in Table 2, and the fixed parameters $A_{p}$, $R_{c}$, $A_{a}$, $\tau_{c}$, $\tau_{h}$, and $\tau_{l}$ of the digital compensator, referring to the acoustic transducer, are set to 5.348 × 10${}^{-11}$ C/Pa, $2\times 10^{10}$ Ω, 3, 4 s, 2.209 s, and 0.01 s, respectively. In the naphtha pipeline, there were 15 abrupt leaks conducted on 20 and 21 November 2013. In the crude oil pipeline, 13 slowly increasing leaks were conducted on 27 October 2016 and 11 December 2017.

### 3.1. Case Study

(1) Slowly increasing leaks

**Figure 11:**
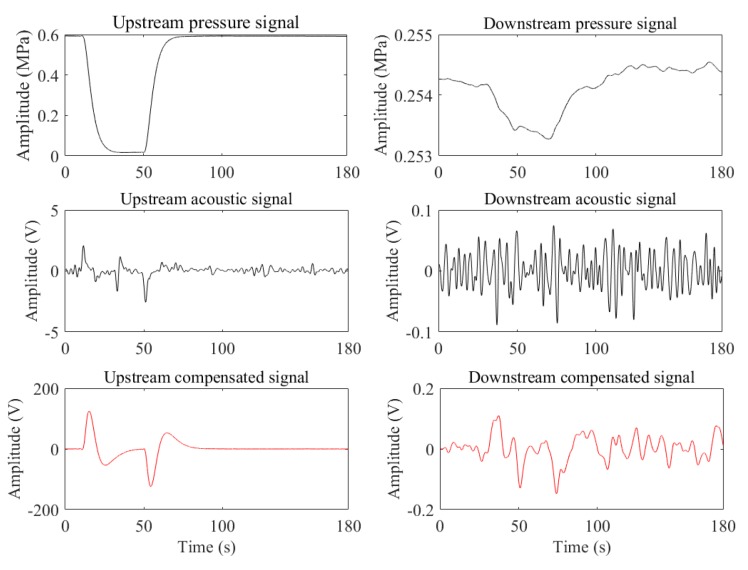
The case of the pressure fluctuation.

Figure 11 is the case of a slowly increasing leak generated when the upstream pressure was steady, and the downstream was fluctuating. Owing to the fluctuation, the identification of the infection points of the downstream pressure signal becomes more difficult, and the acoustic signal of the slowly increasing leak is submerged in the noise. However, the compensated signal ($f_{s}$ = 236 Hz) of the downstream becomes a noticeable pulse, which facilitated its detection. The $f_{s}$ corresponding to the compensated signal of the upstream is 181 Hz. And the SNRs corresponding to the pressure signals, the acoustic signals, and the compensated signal are (upstream: 3.7719 dB, downstream: 0.0131 dB), (upstream: 9.4516 dB, downstream: 0.3084 dB), and (upstream: 20.2370 dB, downstream: 5.8836 dB), respectively. As can be seen, the compensated signals are the best.

**Figure 12:**
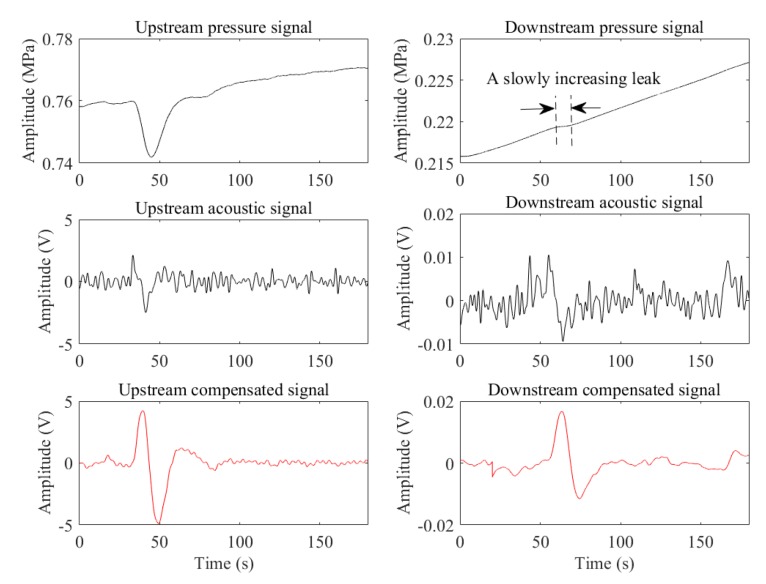
The case of the pressure rising.

Figure 12 illustrates the case of a slowly increasing leak occurred when the upstream pressure was steady, and the downstream was rising. As can be seen, the leak pressure drop propagated from the leak point to the downstream was offset by the upward trend of the downstream pressure. However, as the acoustic transducer and the digital compensator can eliminate static pressure, the leak is noticeable in the acoustic signal and compensated signal (upstream: $f_{s}$ = 116 Hz, downstream: $f_{s}$ = 100 Hz). Moreover, the SNRs of the compensated signals (upstream: 12.1120 dB, downstream: 12.0793 dB) are greater than the acoustic signals (upstream: 5.9677 dB, downstream: 0.8939 dB).

**Figure 13:**
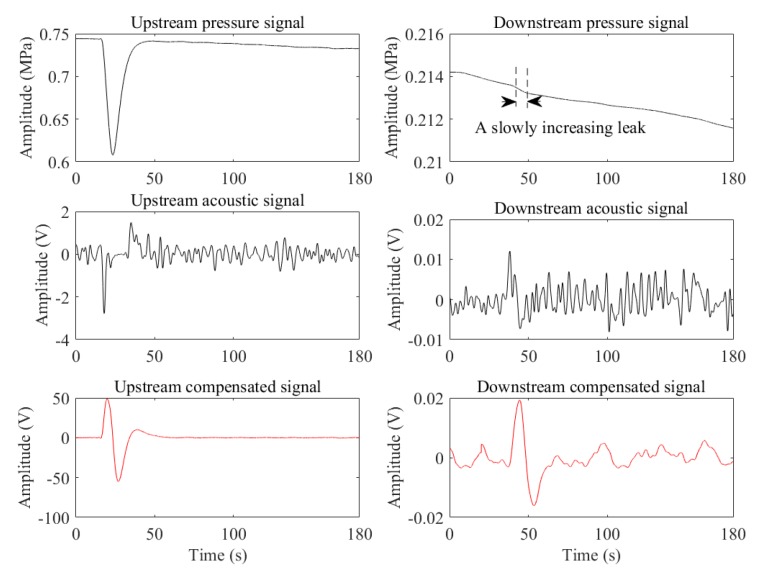
The case of the pressure falling.

Figure 13 shows the case of a slowly increasing leak generated when the upstream pressure was steady and the downstream was falling. The leak pressure drop captured by the pressure transducer of the downstream is hardly distinguishable from the pressure signal. However, the leak in the acoustic signals and the compensated signals (upstream: 184 Hz, downstream: 100 Hz) is more visible, and the corresponding SNRs are (upstream: 9.3254 dB, downstream: 3.4554 dB) and (upstream: 14.7680 dB, downstream: 10.5375 dB), respectively.

(2) Abrupt leaks

**Figure 14:**
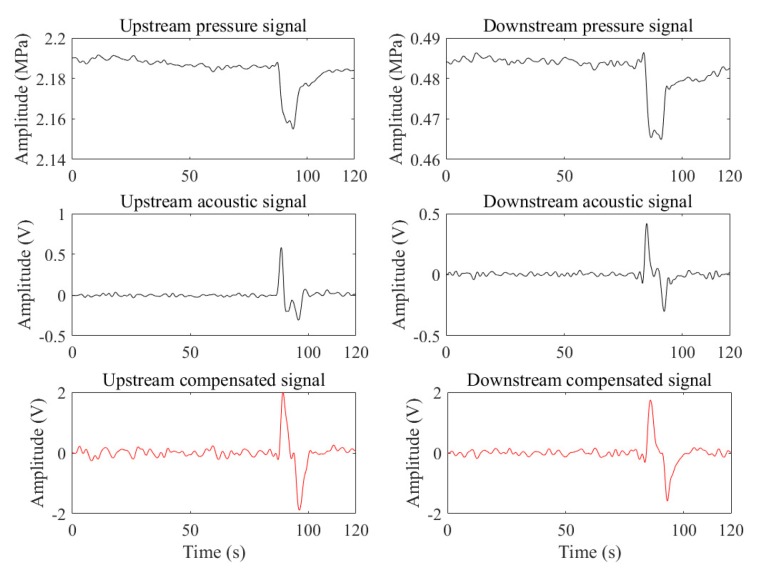
The case of an abrupt leak.

Figure 14 shows the case of an abrupt leak. The SNRs of the pressure signals, the acoustic signals, and the compensated signal are (upstream: 0.2291 dB, downstream: 0.0883 dB), (upstream: 17.8417 dB, downstream: 15.2260 dB), and (upstream: 17.7724 dB, downstream: 15.8009 dB), respectively, and the $f_{s}$ of the upstream and downstream compensated signals is 58 Hz and 80 Hz. From the perspective of the waveform, these signals are obvious. From the perspective of the SNR, the acoustic and compensated signals are greater than the pressure signals, and the acoustic signals and the compensated signals are approximately equal.

In summary, the proposed method is suitable not only for slowly increasing leaks but also for abrupt leaks.

### 3.2. Comparison of Leak Detection and Location

To further prove the validity of the compensated signal, the leak detection and location results of the pressure signals, acoustic signals, and compensated signals are compared. The negative pressure wave method [12] is used to detect the leak pressure signals, and the model-free method [27] is for the leak detection of acoustic signals and compensated signals.

(1) Data set. The data set includes 28 leak samples and 200 normal samples of which 15 abrupt leak samples and 100 normal samples are from the naphtha pipeline, and the rest (13 slowly increasing leak samples and 100 normal samples) are from the crude oil pipeline.

(2) Parameter settings. The alarm thresholds of the negative pressure wave method were set as 10 KPa (naphtha pipeline) and 1 KPa (crude oil pipeline) based on the leak samples (randomly selected) from the data set, and the model-free method does not require parameter setting.

(3) Comparison results. The leak detection and location results are listed in Table 3. For the abrupt leaks from the naphtha pipeline, there is no missing alarm with the acoustic signals and compensated signals, but the pressure signals reached 46.67%. For the slowly increasing leaks from the crude oil pipeline, the compensated signals have no missing alarm, but the pressure signals and acoustic signals were 61.54% and 53.85%, respectively. Additionally, the location error of the pressure signals is the maximum, and the location errors of the acoustic signals and compensated signals are small. Therefore, the performance of compensated signals is better than the others for leak (abrupt leak or slowly increasing leak) detection.

For the normal samples, the false alarm rate of acoustic signals is the lowest, followed by compensated signals and pressure signals. However, the missing alarm rate of acoustic signals and pressure signals is high. After comprehensive consideration of the missing alarm rate and false alarm rate, the compensated signals are the best.

## 4. Conclusions

An enhancement method based on the digital compensator was proposed for the slowly increasing leaks, and the digital compensator is developed according to the advantages of the acoustic transducer. It can not only overcome the effects of static pressure on signals but also converts the slowly decreasing pressure into a noticeable pulse. Based on the adaptive adjustment of the parameter (discretization frequency $f_{s}$), both abrupt and slowly increasing leaks can be enhanced online in real-time. The experimental results indicated the better performance of the compensated signals compared with the pressure signals and the acoustic signals.

## Figures and Tables

**Table 1:** The effect of different parameters on the cutoff frequency result of the model.

Parameters	Values	Cutoff Frequency (rad)	
		Lower	Upper
$f_{s}$ (Hz)	100	0.0058	0.6292
	500	0.0011	0.1299
	1000	0.0005	0.0650
	2000	0.0002	0.0325
*A*(Ω×C/Pa)	7	0.0058	0.6292
	10	0.0058	0.6292
	50	0.0058	0.6292
	100	0.0058	0.6292
$\tau_{c}$ (s)	3	0.0064	0.6298
	20	0.0049	0.6284
	50	0.0049	0.6284
	100	0.0049	0.6284
$\tau_{h}$ (s)	0.5	0.0192	0.6487
	1	0.0103	0.6357
	2	0.0058	0.6292
	3	0.0045	0.6273
$\tau_{l}$ (s)	0.001	0.0059	2.5386
	0.01	0.0058	0.6292
	0.1	0.0052	0.0721
	1	0.0030	0.0120

$A=A_{p}A_{a}R_{c}$, and its unit is Ω×C/Pa because $A_{p}$ is the sensitivity coefficient of the piezoelectric acoustic sensor, and its unit is C/Pa; $A_{a}$ is the gain of the amplifier and is dimensionless, and $R_{c}$ is the feedback resistance of the charge amplifier and its unit is Ω.

**Table 2:** Parameters of the two field pipelines.

Parameters	Pipeline	
	Naphtha	Crude Oil
Total Length (km)	15.511	19.356
Pipe Diameter (mm)	150	219
Leak size (mm)	4 and 8	2
Sound Velocity ($m\cdot s^{-1}$)	1055	900
Upstream Pressure (MPa)	2.18	0.80
Downstream Pressure (MPa)	0.48	0.1
Leak Location from upstream (km)	9.476	8.5
Sampling Frequency (Hz)	50	100
Pressure Sensor Type	STG74S of Honeywell	
Acoustic Sensor Type	Dynamic pressure sensor	
Sensor Location	Installed on both ends of the pipeline	

**Table 3:** Leak detection and location results of the compensated signals, acoustic signals, and pressure signals.

Pipeline	Signal	Missing Alarm Rate (%)	False Alarm Rate (%)	Maximum Relative Error (%)
Naphtha	**Compensated signal**	**0**	**0**	**0.29**
	Acoustic signal	0	0	0.26
	Pressure Signal	46.67	0	9.03
Crude oil	**Compensated signal**	**0**	**2**	**0.28**
	Acoustic signal	61.54	0	0.30
	Pressure Signal	53.85	5	10.68

Missing alarm rate= (Total leak number − Correct leak alarm number)/Total leak number%. False alarm rate = False alarm number/Total normal number%. Relative error = |Leak location-Pipeline length|/Pipeline length%.

## Appendix A

To prove:

(A1) $$\begin{array}{ll} \left|H(e^{j\omega })\right| & =20\mathrm{lg}\left(A\tau_{c}\tau_{h}^{2}\right)+10\mathrm{lg}\frac{2f_{s}\tau_{c}\tau_{h}\tau_{l}\tan\frac{\omega }{2}}{\tau_{h}^{2}\tau_{l}^{2}+{\left(2f_{s}\tau_{c}\tau_{h}\tau_{l}\tan\frac{\omega }{2}\right)}^{2}}\\ & +10\mathrm{lg}\frac{2f_{s}\tau_{c}\tau_{h}\tau_{l}\tan\frac{\omega }{2}}{\tau_{c}^{2}\tau_{l}^{2}+{\left(2f_{s}\tau_{c}\tau_{h}\tau_{l}\tan\frac{\omega }{2}\right)}^{2}}+20\mathrm{lg}\frac{2f_{s}\tau_{c}\tau_{h}\tau_{l}\tan\frac{\omega }{2}}{\tau_{c}^{2}\tau_{h}^{2}+{\left(2f_{s}\tau_{c}\tau_{h}\tau_{l}\tan\frac{\omega }{2}\right)}^{2}} \end{array}$$

**Proof.** According to Equation (14), the $\left|H(e^{j\omega })\right|$ is:

(A2) $$\left|H(e^{j\omega })\right|=20\mathrm{lg}\left(\frac{16A\tau_{h}f_{s}^{2}\sin^{2}\omega \left(\sin^{2}\omega +\cos^{2}\omega \right)}{\prod_{m=1}^{4}\sqrt{{\left[\left(2f_{s}\tau_{m}+1\right)\cos\omega -\left(2f_{s}\tau_{m}-1\right)\right]}^{2}+{\left[(2f_{s}\tau_{m}+1)\sin\omega \right]}^{2}}}\right)$$

Since

(A3) $$\sin\omega =\frac{2\tan\frac{\omega }{2}}{1+\tan^{2}\frac{\omega }{2}},\cos\omega =\frac{1-\tan^{2}\frac{\omega }{2}}{1+\tan^{2}\frac{\omega }{2}},\sin^{2}\omega +\cos^{2}\omega =1$$

Equation (A2) can be rewritten as:

(A4) $$\begin{array}{ll} \left|H(e^{j\omega })\right| & =20\mathrm{lg}\left(\frac{16A\tau_{h}f_{s}^{2}\sin^{2}\omega }{\prod_{m=1}^{4}\sqrt{{\left[\left(2f_{s}\tau_{m}+1\right)\cos\omega -\left(2f_{s}\tau_{m}-1\right)\right]}^{2}+{\left[(2f_{s}\tau_{m}+1)\sin\omega \right]}^{2}}}\right)\\ & =20\mathrm{lg}\left(A\tau_{h}\prod_{m=1}^{4}\sqrt{\frac{2f_{s}\sin\omega }{\left(4f_{s}^{2}\tau_{m}^{2}+1\right)-\left(4f_{s}^{2}\tau_{m}^{2}-1\right)\cos\omega }}\right)\\ & =20\mathrm{lg}\left(A\tau_{h}\prod_{m=1}^{4}\sqrt{\frac{2f_{s}\frac{2\tan\frac{\omega }{2}}{1+\tan^{2}\frac{\omega }{2}}}{\left(4f_{s}^{2}\tau_{m}^{2}+1\right)-\left(4f_{s}^{2}\tau_{m}^{2}-1\right)\frac{1-\tan^{2}\frac{\omega }{2}}{1+\tan^{2}\frac{\omega }{2}}}}\right)\\ & =20\mathrm{lg}\left(A\tau_{h}\prod_{m=1}^{4}\sqrt{\frac{2f_{s}\tan\frac{\omega }{2}}{1+4f_{s}^{2}\tau_{m}^{2}\tan^{2}\frac{\omega }{2}}}\right) \end{array}$$

According to the logarithmic algorithm, Equation (A4) can be decomposed into:

(A5) $$\begin{array}{ll} \left|H(e^{j\omega })\right|= & 20\mathrm{lg}\left(A\tau_{h}\right)+10\mathrm{lg}\frac{2f_{s}\tan\frac{\omega }{2}}{1+4f_{s}^{2}\tau_{c}^{2}\tan^{2}\frac{\omega }{2}}\\ & +10\mathrm{lg}\frac{2f_{s}\tan\frac{\omega }{2}}{1+4f_{s}^{2}\tau_{h}^{2}\tan^{2}\frac{\omega }{2}}+20\mathrm{lg}\frac{2f_{s}\tan\frac{\omega }{2}}{1+4f_{s}^{2}\tau_{l}^{2}\tan^{2}\frac{\omega }{2}} \end{array}$$

The last three items on the right side of the above equation are processed as follows:

(A6) $$10\mathrm{lg}\frac{2f_{s}\tan\frac{\omega }{2}}{1+4f_{s}^{2}\tau_{c}^{2}\tan^{2}\frac{\omega }{2}}=10\mathrm{lg}\frac{2f_{s}\tau_{c}\tau_{h}\tau_{l}\tan\frac{\omega }{2}}{\tau_{h}^{2}\tau_{l}^{2}+{\left(2f_{s}\tau_{c}\tau_{h}\tau_{l}\tan\frac{\omega }{2}\right)}^{2}}-10\mathrm{lg}\frac{\tau_{c}}{\tau_{h}\tau_{l}}$$

(A7) $$10\mathrm{lg}\frac{2f_{s}\tan\frac{\omega }{2}}{1+4f_{s}^{2}\tau_{h}^{2}\tan^{2}\frac{\omega }{2}}=10\mathrm{lg}\frac{2f_{s}\tau_{c}\tau_{h}\tau_{l}\tan\frac{\omega }{2}}{\tau_{c}^{2}\tau_{l}^{2}+{\left(2f_{s}\tau_{c}\tau_{h}\tau_{l}\tan\frac{\omega }{2}\right)}^{2}}-10\mathrm{lg}\frac{\tau_{h}}{\tau_{c}\tau_{l}}$$

(A8) $$20\mathrm{lg}\frac{2f_{s}\tan\frac{\omega }{2}}{1+4f_{s}^{2}\tau_{l}^{2}\tan^{2}\frac{\omega }{2}}=20\mathrm{lg}\frac{2f_{s}\tau_{c}\tau_{h}\tau_{l}\tan\frac{\omega }{2}}{\tau_{c}^{2}\tau_{h}^{2}+{\left(2f_{s}\tau_{c}\tau_{h}\tau_{l}\tan\frac{\omega }{2}\right)}^{2}}-20\mathrm{lg}\frac{\tau_{l}}{\tau_{c}\tau_{h}}$$

Hence, Equation (A5) is

(A9) $$\begin{array}{ll} \left|H(e^{j\omega })\right| & =20\mathrm{lg}\left(A\tau_{h}\right)-10\mathrm{lg}\frac{\tau_{c}}{\tau_{h}\tau_{l}}-10\mathrm{lg}\frac{\tau_{h}}{\tau_{c}\tau_{l}}-20\mathrm{lg}\frac{\tau_{l}}{\tau_{c}\tau_{h}}+10\mathrm{lg}\frac{2f_{s}\tau_{c}\tau_{h}\tau_{l}\tan\frac{\omega }{2}}{\tau_{h}^{2}\tau_{l}^{2}+{\left(2f_{s}\tau_{c}\tau_{h}\tau_{l}\tan\frac{\omega }{2}\right)}^{2}}\\ & +10\mathrm{lg}\frac{2f_{s}\tau_{c}\tau_{h}\tau_{l}\tan\frac{\omega }{2}}{\tau_{c}^{2}\tau_{l}^{2}+{\left(2f_{s}\tau_{c}\tau_{h}\tau_{l}\tan\frac{\omega }{2}\right)}^{2}}+20\mathrm{lg}\frac{2f_{s}\tau_{c}\tau_{h}\tau_{l}\tan\frac{\omega }{2}}{\tau_{c}^{2}\tau_{h}^{2}+{\left(2f_{s}\tau_{c}\tau_{h}\tau_{l}\tan\frac{\omega }{2}\right)}^{2}}\\ & =20\mathrm{lg}\left(A\tau_{c}\tau_{h}^{2}\right)+10\mathrm{lg}\frac{2f_{s}\tau_{c}\tau_{h}\tau_{l}\tan\frac{\omega }{2}}{\tau_{h}^{2}\tau_{l}^{2}+{\left(2f_{s}\tau_{c}\tau_{h}\tau_{l}\tan\frac{\omega }{2}\right)}^{2}}\\ & +10\mathrm{lg}\frac{2f_{s}\tau_{c}\tau_{h}\tau_{l}\tan\frac{\omega }{2}}{\tau_{c}^{2}\tau_{l}^{2}+{\left(2f_{s}\tau_{c}\tau_{h}\tau_{l}\tan\frac{\omega }{2}\right)}^{2}}+20\mathrm{lg}\frac{2f_{s}\tau_{c}\tau_{h}\tau_{l}\tan\frac{\omega }{2}}{\tau_{c}^{2}\tau_{h}^{2}+{\left(2f_{s}\tau_{c}\tau_{h}\tau_{l}\tan\frac{\omega }{2}\right)}^{2}} \end{array}$$

Therefore,

(A10) $$\begin{array}{ll} \left|H(e^{j\omega })\right| & =20\mathrm{lg}\left(A\tau_{c}\tau_{h}^{2}\right)+10\mathrm{lg}\frac{2f_{s}\tau_{c}\tau_{h}\tau_{l}\tan\frac{\omega }{2}}{\tau_{h}^{2}\tau_{l}^{2}+{\left(2f_{s}\tau_{c}\tau_{h}\tau_{l}\tan\frac{\omega }{2}\right)}^{2}}\\ & +10\mathrm{lg}\frac{2f_{s}\tau_{c}\tau_{h}\tau_{l}\tan\frac{\omega }{2}}{\tau_{c}^{2}\tau_{l}^{2}+{\left(2f_{s}\tau_{c}\tau_{h}\tau_{l}\tan\frac{\omega }{2}\right)}^{2}}+20\mathrm{lg}\frac{2f_{s}\tau_{c}\tau_{h}\tau_{l}\tan\frac{\omega }{2}}{\tau_{c}^{2}\tau_{h}^{2}+{\left(2f_{s}\tau_{c}\tau_{h}\tau_{l}\tan\frac{\omega }{2}\right)}^{2}} \end{array}$$

 □
